# *In-situ* characterization of nanoparticle beams focused with an aerodynamic lens by Laser-Induced Breakdown Detection

**DOI:** 10.1038/srep15696

**Published:** 2015-10-26

**Authors:** F.-A. Barreda, C. Nicolas, J.-B. Sirven, F.-X. Ouf, J.-L. Lacour, E. Robert, S. Benkoula, J. Yon, C. Miron, O. Sublemontier

**Affiliations:** 1 CEA, DSM/IRAMIS/NIMBE-UMR 3685, F-91191 Gif-sur-Yvette, France; 2 Synchrotron SOLEIL, L’Orme des Merisiers, Saint-Aubin BP 48, F-91192 Gif-sur-Yvette Cedex, France; 3 CEA, DEN, Department of Physical Chemistry, F-91191 Gif-sur-Yvette, France; 4 Institut de Radioprotection et de Sûreté Nucléaire (IRSN), BP68, F-91192 Gif-sur-Yvette Cedex, France; 5 UMR 6614 CORIA, CNRS, Université et INSA de Rouen, BP12, F-76801 Saint-Etienne du Rouvray Cedex, France; 6 Extreme Light Infrastructure - Nuclear Physics (ELI-NP), "Horia Hulubei" National Institute for Physics and Nuclear Engineering, 30 Reactorului Street, RO-077125 Măgurele, Jud. Ilfov, Romania

## Abstract

The Laser-Induced Breakdown Detection technique (LIBD) was adapted to achieve fast *in-situ* characterization of nanoparticle beams focused under vacuum by an aerodynamic lens. The method employs a tightly focused, 21 μm, scanning laser microprobe which generates a local plasma induced by the laser interaction with a single particle. A counting mode optical detection allows the achievement of 2D mappings of the nanoparticle beams with a reduced analysis time thanks to the use of a high repetition rate infrared pulsed laser. As an example, the results obtained with Tryptophan nanoparticles are presented and the advantages of this method over existing ones are discussed.

Laser-based techniques (e.g. light scattering, laser-induced plasma or Raman-based spectroscopy), for *in-situ* and real-time analysis of nanoparticles have been applied for different applications, such as process control or effluent waste monitoring in atmospheric or in environmental sciences^1^,^2^,^3^,^4^. The ease of their implementation, as well as the ability for remote, *in-situ* and real-time analysis make these techniques perfectly suitable when sample handling should be minimized or when sampling is not directly possible (radioactive elements or under vacuum samples for instance). In addition, most of these methods are non-invasive and almost non-destructive as only a tiny fraction of the total amount of particles is used for the characterization.

Among these laser-based techniques, LIBD is a very sensitive method, well known for the determination of colloid size distributions in aqueous solutions^5^,^6^,^7^. In this technique, a pulsed laser beam is tightly focused on particles and the induced breakdown is then detected either using an acoustic method (piezo-receiver) for monitoring the plasma shockwave^8^ or an optical method for collecting the emitted light^9^. In our case, the light emitted by the plasma is collected without spectral analysis, resulting in an enhanced sensitivity compared to the classical Laser Induced Breakdown Spectroscopy technique (LIBS). It was demonstrated that LIBD is able to detect nanoparticles with sizes as low as 5 nm^10^ and concentrations inferior to 10^6^ particles/cm^3^, whereas conventional light scattering methods require more drastic experimental conditions such as orders of magnitude larger particle densities (above 10^10^ particles/cm^3^ for the detection of the same size range^11^) and/or particles in the form of fractal aggregates^12^.

The aim of the present work is to adapt the LIBD technique in order to characterize nanoparticle beams generated with an Aerodynamic Lens System (ALS) allowing the transfer of nanoparticles from atmospheric pressure to secondary vacuum. Aerodynamic particle focusing is accomplished by successive compression and expansion of a carrier gas through a series of coaxial orifices with different diameters. Owing to inertia effects, nanoparticles are progressively separated from the gas streamlines and focused along the lens symmetry axis. Since their original development by Peter Mc Murry^13^,^14^ in 1995, several other research groups studied these aerodynamic lenses in order to characterize and optimize their performances^15^,^16^,^17^,^18^,^19^. These systems are commonly used as introduction tool for aerosol mass spectrometers^20^,^21^,^22^,^23^ due to their ability to focus, with high transmission efficiency, broad size distributions of nanoparticles into a sub-millimeter sized beam. The ALS is an essential tool for chemical studies of particles’ properties such as reactivity, where it is important for the sample to be free from any interaction with a substrate. More recently, such systems have been also used to obtain an efficient interaction between nano-objects and radiation sources such as synchrotrons^24^, free electron lasers^25^,^26^ or conventional lasers^27^ for a diversified range of scientific studies. Although ALS have been widely characterized by numerical simulations^13^,^18^,^28^,^29^, the experimental attempts to systematically evaluate their focusing properties are sparse^19^,^22^. However, the characterization of nanoparticle beams is a key issue, as their dimensions have a direct impact on the interaction efficiency with a specific probe. For synchrotron radiation studies for instance, it is important to generate a nanoparticle beam with dimensions in the same range as the typical synchrotron beam size *i.e.* around 200 μm in the case of PLEIADES beamline at the Synchrotron SOLEIL facility where this development was performed.

We present here a technique derived from LIBD for *in-situ* probing of nanoparticle beams under vacuum. An experimental setup, including a scanning laser microprobe, has been developed to perform a direct characterization of the aerodynamic lens focusing via a 2D mapping of the nanoparticle beam produced by an ALS. The principle is based on the breakdown occurrence only in the presence of at least one nanoparticle in the laser’s focal volume. The laser focusing system is designed to allow single particle detection, and the use of a high repetition rate pulsed laser (up to 25 kHz), never tested for LIBD measurements, permits fast nanoparticles sampling.

For testing purposes, beam profile measurements were performed on Tryptophan (C_11_H_12_N_2_O_2_) nanoparticles, a molecule commonly used in the framework of biomolecular studies on PLEIADES beamline at the Synchrotron SOLEIL facility. The nanoparticles were generated by a continuous output atomizer (N°3076, TSI) from a 2 g/l aqueous solution of Tryptophan using air as carrier gas. Tryptophan nanoparticles, initially embedded in water droplets, are dried up by passing through two diffusion dryers. The particle size distribution and number density are measured with a commercial differential mobility analyzer coupled to a condensation particle counter (DMA 3081 & CPC 3786, TSI Inc.). A log-normal distribution of Tryptophan nanoparticles is thus produced with a mode diameter of 136 nm, a geometric standard deviation of 2.0 and a total concentration of 2.5 × 10^6^ particles/cm^3^.

Aerosols are then introduced into the vacuum chamber using the ALS developed at the PLEIADES beamline at the SOLEIL Synchrotron radiation facility (Fig. 1(a)), described in detail in reference 30. The lens geometry is based on the design reported by Zhang *et al.*^17^,^18^,^17^,^31^ consisting of a limiting orifice (200 μm) and five thin-plate orifices separated by chambers and ended by a 3-mm diameter accelerating nozzle. The lens system is pumped by two, high-capacity turbomolecular pumps (2300 l/s) ensuring in the nanoparticles source chamber pressures below 10^−6^ mbar before nanoparticles’ introduction. At the lens output, the aerosol beam passes through a 1 mm skimmer for removal of the main gas stream before entering the interaction chamber (Fig. 1(b)), which is pumped by an additional turbomolecular pump (1000 l/s). The laser microprobe is installed at 51 mm from the skimmer orifice, and the distance between the skimmer and the lens output is 3 mm. In these conditions, the operating pressures in the interaction chamber are in the 10^−4^ mbar range when nanoparticles are introduced.

The excitation source is a Q-switched rod-type fiber laser with an active gain medium consisting of an ytterbium-doped photonic crystal fiber^32^ (Boreas HE-IR20, Eolite). The laser emits in the infrared (λ = 1030 nm) with a nominal pulse width varying from 9 to 20 ns for repetition rates between 1 and 25 kHz, respectively. At the cavity output, the maximum pulse energy is 2 mJ and the maximum average power is 20 W with an excellent beam profile (beam quality factor M^2^ close to 1). The laser beam is guided and introduced inside the interaction chamber using an optical fiber (numerical aperture N.A. = 0.05, core diameter = 105 μm, length > 6 m), which ensures a good quality beam allowing suitable irradiances for the plasma formation. The fiber connector is water-cooled to avoid fiber degradation during continuous use at high repetition rates. The beam is collimated with a 100-mm focal length lens. A 45° dichroic mirror reflects the beam toward a microscope objective (N.A. = 0.25, working distance W.D. = 15 mm, magnification = 10) which focuses the laser beam. The whole optical assembly is mounted on two computer-controlled motorized translation stages, with the translation axes perpendicular to each other (Fig. 1(b)) so that the laser microprobe permits 2D scanning of the nanoparticle beam. The laser energy at the target is monitored by a wattmeter located outside of the vacuum chamber using a 30-mm focal length lens to collimate the beam after the interaction region. The plasma emission is collected with a photomultiplier tube (PMT R212, Hamamatsu) equipped with a 0° cavity laser mirror to filter the light from the laser and is located perpendicularly to the laser axis. During 2D scans of the nanoparticles’ beam, the optical emission signal is monitored in counting mode from each plasma event at the different laser positions within the beam and hence, only events with a response exceeding the noise threshold are counted during the integration time. The advantage of this method is to overcome fluctuations in signal intensity, the number of events thus being directly related to the density of nanoparticles at different locations within the nanoparticle beam, while the measurement uncertainty is well characterized by a Poisson distribution. The overall setup is presented in Fig. 1.

For the development of the laser microprobe, a tightly focused beam is pre-requisite in order to have a sufficient spatial resolution to measure sub-millimeter wide nanoparticles’ beams. As the laser beam characteristics have a strong impact on its focusing, and hence on the laser/matter interaction^33^, the laser beam profile at the optical fiber output has been characterized. A 1-m focal length lens is used to image the beam waist at the fiber output on a CCD camera (BeamStar FX 50, Ophir) and the pulse energy is attenuated with appropriate neutral density filters to avoid saturation of the camera. The spatial intensity distribution within the beam is monitored for different laser repetition rates at the maximum power available (Fig. 2).

A top-hat-like profile is obtained with a beam quality factor M^2^ between 7.6 and 9.5 depending on repetition rate. The beam divergence is 50 mrad, as expected according to the optical fiber properties. All these parameters are used to theoretically model the dimensions of the beam waist in the focal plane of our optical setup. Note that hot spots (in red) are present in the beam spatial profiles for repetition rates larger or equal to 5 kHz. This may induce a non-negligible bias on the real laser volume of interaction that have to be taken into account for further estimation of the particle density in the nanoparticle beam. In order to check the laser spot size, a steel target is placed in the laser microprobe focal plane and the resulting craters are measured with a 3D white-light interferometric profilometer (ContourGT-I, BRUKER). This provides a representative measurement of the beam waist in our experimental conditions, as the target nature does not significantly affect the crater size for a top-hat laser beam. Figure 3 shows a typical crater obtained from a single laser shot with the developed microprobe. The center of the obtained profile reflects the spatial distribution of the laser intensity, while the significant broadening close to the surface is induced by erosion due to plasma/surface interaction at high laser energy. Calculations based on Gaussian beams propagation predict a focal diameter of 21.4 ± 2.3 μm and the measurements on the steel target show a mean diameter of the central part of the crater of 20.8 ± 1.6 μm for the different repetition rates. The good agreement between these values confirms that the laser setup is fully characterized.

With this optical configuration, we introduce the effective volume *V*_*eff*_, defined as the portion of the interaction region where the laser irradiance is higher or equal to the breakdown threshold from a particle. Figure 4 shows the breakdown probability as a function of the laser irradiance. From this figure, a breakdown threshold irradiance of 5.7 GW/cm^2^ is estimated for 130 nm tryptophan nanoparticles. This value is quite high in comparison with what is usually observed for solid targets. This can be explained both by the low density of the biological material constituting tryptophan and by its particulate nature. Note that the breakdown threshold irradiance of water droplets was previously measured at 6 GW/cm^2^ at 1064 nm^34^.

The measurements reported in Fig. 4 where obtained from different laser configurations listed in Table 1. For different repetition rates between 5 and 25 kHz, the energy per pulse and the M^2^ are different, leading to different irradiances and different effective volumes. In our experimental conditions, this corresponds to an average particle number *μ* in the effective volume between 2.8 × 10^4^ and 7.1 × 10^4^ μm^3^. Assuming Poisson statistics, the particle sampling rate (PSR), defined as the percentage of laser pulses expected to sample at least one particle, is given by the expression^35^: *PSR* = (1 − *e*^*−μ*^). Taking into account the particle concentration measured after atomization of the suspension, and considering that only 10% of the produced particles are introduced in the ALS, we can estimate the density of particles in the laser focal volume. With a volume flow rate of 0.3 l/s in the ALS and a particle velocity of 250 m/s at the ALS outlet in a 200 μm nanoparticle beam diameter in the interaction region, we find an average particle density around 1.6 × 10^5^ particles/cm^3^ in the interaction region. In these conditions, the maximum expected PSR is 1.1%. The probability of detecting more than one particle in the focal volume is then perfectly negligible, regardless of the laser conditions. Consequently, it is possible to monitor the signal from the PMT in counting mode as we achieve single-particle sampling and thus, profiles of the nanoparticle beam can be determined with the laser microprobe developed in this work.

The experimental PSR is much lower than the expected one in all laser configurations. This could be partially explained by a possible agglomeration process that could occur within the beam and by particles loss at the limiting orifice of the ALS. Nevertheless, this difference is more likely due to an underestimation of the breakdown threshold from Fig. 4. If we consider the laser spatial profiles shown in Fig. 2, the red spots show higher local intensities in the focal volume for repetition rates higher or equal to 5 kHz. If the breakdown threshold is reached only in the corresponding local higher intensity regions, then the effective volume is highly overestimated, and could easily be a factor of ten less than what was calculated assuming a homogeneous spatial profile. The presence of a thin solvent layer around the particles due to incomplete drying can also contribute to a weaker nanoparticle-laser interaction resulting in lower PSR. This can be the case for hydrophilic material particles like TiO_2_^36^. Work is currently under progress to study in more detail breakdown thresholds on nanoparticles using this setup.

The choice of the laser configuration for nanoparticle beam characterization was made on the criteria of maximum experimental signal to noise ratio, obtained for a repetition rate of 20 kHz (see Table 1). It should also be noted that, at this repetition rate, the time between two consecutive laser shots is 50 μs, whereas the plasma emission duration measured with an oscilloscope does not overcome 250 ns. Consequently, the plasma has already vanished when the next laser shot occurs, allowing the use of this high laser repetition rate. Compared to classical LIBS analysis, where plasmas can last several microseconds, plasma lifetimes obtained in our experimental conditions are short due to its generation under vacuum. Indeed, low pressure (below 1 mbar) causes an extremely rapid plasma expansion with decreasing collisions, leading to a faster decay of excited species and hence plasma lifetimes shorter than 1 μs^37^,^38^. Besides, focusing conditions are designed to achieve less than one nanoparticle in the focal plane per laser shot, thus the vaporized matter quantity is strongly reduced compared to bulk or microparticles analysis explaining the short plasma duration observed.

The 2D mapping is performed on a Tryptophan nanoparticle beam with an average laser power of 10.7 W at 20 kHz, corresponding to a laser average irradiance of 11.6 GW/cm^2^ on the target. The number of events is recorded for a counting time of 10 s per position for the 1D profile (Fig. 5(a)) and 5 s for the 2D mapping (Fig. 5(b)). Signal is also monitored in absence of nanoparticles for comparison, showing a signal-to-noise ratio above 150. A scan with only water in the atomizer connected to the diffusion dryers was also performed and a similar flat profile was observed. From the number of events determined for each X and Z position, a 2D visualization of the beam can be obtained (Fig. 5(b)). Thanks to the high laser repetition rate, 1D profiles can be performed in only a few minutes and 2D mappings in approximately 20 minutes. With a laser repetition rate of 20 Hz commonly employed in LIBD setups, the same cartography would require 14 days acquisition time. 2D data are smoothed with the LOESS function (Locally Weighted Scatterplot Smoothing) using the Igor Pro software (Wavemetrics Inc.) for an average representation of the spatial density distribution. As can be seen in Fig. 5(a), the fitted beam profile presents a Lorentzian shape as already observed for nanoparticle beams produced with an ALS^39^. The full width at half maximum (FWHM) is determined to be 70 μm ± 6 μm and the total width, defined so that 90% of the maximum intensity was contained in the beam profile, is found to be 211 μm ± 22 μm in average from the experimental profiles. The beam profile was also determined with 10 and 25 kHz repetition rates showing no significant changes. That indicates that the beam profile is accurately and consistently determined with the laser microprobe, independently of the repetition rate and the laser energy used.

In order to compare the reliability of these results with the real beam dimensions, Tryptophan nanoparticles were also deposited on a glass substrate inserted inside the vacuum chamber near the interaction region, to be representative of the diameter measured with the laser. Images of the deposits were taken using an optical microscope and the nanoparticles’ beam dimensions were measured with the ImageJ open access software^40^. Although the diameters observed on the glass substrate are of the same order of magnitude as the ones determined with our laser microprobe, it was noticed that the beam dimensions on the substrate increased by almost a factor of 3 between 2 and 10 min deposition time (70–220 μm). In addition, the determination of the beam diameters from the microscope image depends on the method chosen by the operator to define the mean diameter, increasing the uncertainty on the measurement. This illustrates the serious drawbacks of this simple method and points out the advantage of LIBD for *in-situ* characterization of the nanoparticle beams. In addition, when compared to the method based on measurements with an electrometer^19^, which requires the use of a knife edge beam stop and the knowledge of the electrical charge state, the laser microprobe can be applied to non charged nanoparticles and provides beam visualization in 3D without a need for particles deposition. The method is also highly relevant in the case of small (<150 nm), non-agglomerated and diluted nanoparticles in comparison with light scattering techniques^22^ that are not suited for so rarefied media and do not provide a laser probe size in the tens of micrometers range (generally limited to hundreds of micrometers).

An innovative and promising application of the laser-induced plasma technique based on the use of a laser microprobe (21 μm) for 2D mappings of nanoparticle beams in vacuum is presented. High repetition rate LIBD is proposed for fast, direct determination of nanoparticle beam profiles at the outlet of an aerodynamic lens system. The proof-of-principle is provided using a beam of 136 nm diameter Tryptophan nanoparticles focused with an aerodynamic lens system whose design has been inspired by Zhang’s lens geometry. Our results reveal a Lorentzian beam profile with a FWHM of 70 μm ± 6 μm in average for Tryptophan. The experimental particle sampling rate appears to be much lower than expected. We interpret this difference mainly by hot spots in the laser spatial profile that could be responsible for a significant overestimation of the real effective volume of interaction.

## Additional Information

**How to cite this article**: Barreda, F.-A. *et al.* *In-situ* characterization of nanoparticle beams focused with an aerodynamic lens by Laser-Induced Breakdown Detection. *Sci. Rep.* **5**, 15696; doi: 10.1038/srep15696 (2015).

## Figures and Tables

**Figure 1:**
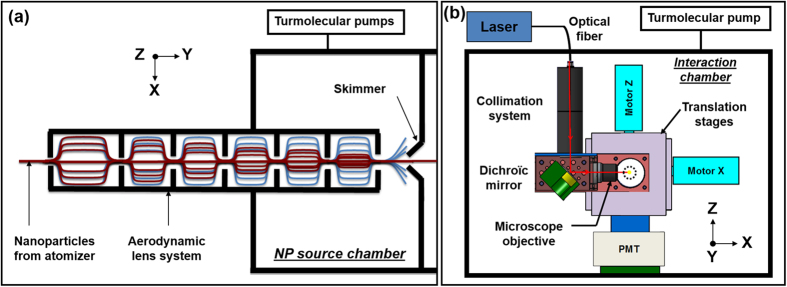
(**a**) Aerodynamic Lens System for the nanoparticles (NP) beam generation and (**b**) laser microprobe setup. Note that (**a**) is represented in the XY plane whereas (**b**) is represented in the XZ plane. In the NP source chamber (**a**), the red lines refer to the nanoparticles beam and the blue lines refer to the carrier gas removed by the skimmer before NP introduction into the interaction chamber (**b**). On the LIBD setup (**b**), the laser beam trajectory is drawn in red and, in the interaction region (dotted line), the microplasma induced when a particle enters the focal volume is indicated in yellow. The system for laser energy measurement was not drawn for the sake of clarity.

**Figure 2:**
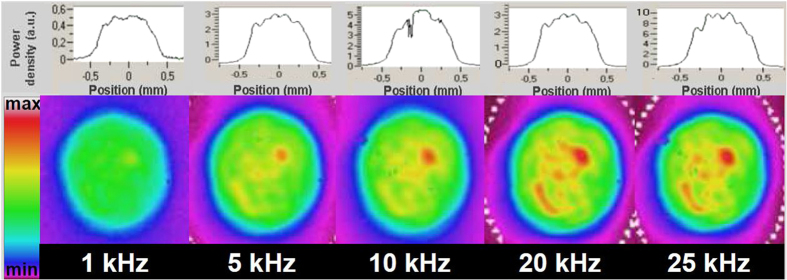
Laser beam profiles for different repetition rates.

**Figure 3:**
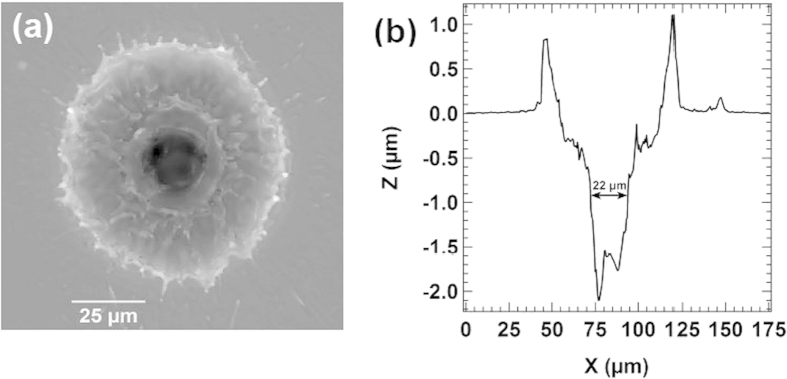
(**a**) Image and (**b**) profile of a single-shot crater produced by the laser microprobe on a steel target with an irradiance of 20 GW/cm^2^ and a repetition rate of 10 kHz.

**Figure 4:**
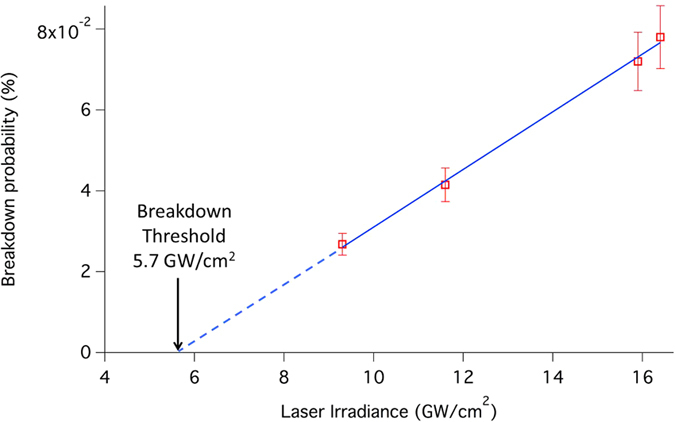
Breakdown probability as a function of laser irradiance. The deduced breakdown threshold is estimated at 5.7 GW/cm^2^.

**Figure 5:**
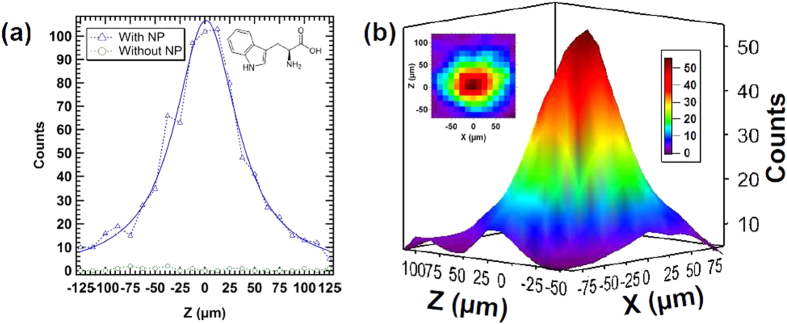
(**a**) 1D profile and (**b**) 2D mapping with 3D visualization of the Tryptophan nanoparticles beam performed with the laser microprobe at an average power of 10.7 W and a repetition rate of 20 kHz. The blue solid line on the 1D profile represents the Lorentzian fit used for beam width determinations.

**Table 1:** Laser parameters listed with corresponding expected and measured experimental parameters.

Repetition rate (kHz)	Energy per pulse (mJ)	Irradiance (GW/cm^2^)	V_eff_(μm^3^)	Expected PSR (%)	Measured PSR (%)	Measured count-rate (cts in 10 s)
5	0.76	15.9	7.1 × 10^4^	1.1	0.07	36
10	0.78	16.4	5.9 × 10^4^	0.9	0.08	78
**20**	**0.55**	**11.6**	**3.8** × **10**^4^	**0.6**	**0.04**	**83**
25	0.44	9.3	2.8 × 10^4^	0.4	0.03	67
